# Clinical Characteristics of Acute Appendicitis in Pregnancy: 10-Year Experience at a Single Institution in South Korea

**DOI:** 10.3390/jcm12093277

**Published:** 2023-05-04

**Authors:** Yun Suk Choi, Ji Hyun Seo, Jin Wook Yi, Yun-Mee Choe, Yoon Seok Heo, Sun Keun Choi

**Affiliations:** Department of Surgery, Inha University Hospital, Inha University College of Medicine, Jung-gu, Inchon 22332, Republic of Korea; yunsukki@gmail.com (Y.S.C.); jhseo86@gmail.com (J.H.S.); jinwook.yi@inha.ac.kr (J.W.Y.); gsmee@inha.ac.kr (Y.-M.C.); gshur@inha.ac.kr (Y.S.H.)

**Keywords:** acute appendicitis, pregnant women, appendectomy

## Abstract

Background: Acute appendicitis is the most common cause of non-obstetric surgical disease in pregnant women. The diagnosis and treatment of appendicitis during pregnancy are very important because it can cause life-threatening morbidity to the fetus and mother. We evaluated the clinical characteristics of acute appendicitis in pregnant women. Methods: We retrospectively reviewed a medical database that included patients who underwent surgery for acute appendicitis at our hospital from January 2013 through December 2022. We compared non-pregnant women of reproductive age with pregnant women. We classified the pregnant women according to gestational age. Result: A total of 828 patients were reproductive-aged women between 15 and 44 years old. There were 759 non-pregnant patients and 69 pregnant patients. ASA (American Society of Anesthesiologists) physical status classes were significantly higher and hospital stays were significantly longer in the pregnant group. There was no significant intergroup difference in terms of the proportions of complicated appendicitis, extended surgery, or complications. When the enrolled pregnant women were divided into three subgroups according to gestational age, the mean operation time was longest in the third-trimester subgroup. There were no differences among the subgroups in terms of the proportions of complicated appendicitis, extended surgery, or complications, nor were there differences among the subgroups in terms of laboratory findings. Preterm labor and stillbirth occurred in two pregnant women with complicated appendicitis in the second trimester. Conclusion: Immediate surgical treatment should be strongly considered in pregnant women with appendicitis. Efforts for more accurate diagnosis are needed for pregnant women with appendicitis.

## 1. Introduction

Acute appendicitis is the most common disease requiring emergency surgery among abdominal organ diseases. The lifetime incidence of acute appendicitis has been reported to be 8.6% among men and 6.7% among women, and it is known to occur in one out of every 15 people [1,2]. Several studies have investigated non-surgical treatment for acute appendicitis, but the gold standard is surgical appendectomy [3,4,5]. If non-operative treatment fails, there is increased risk of morbidity, such as that associated with the higher probability of open surgery and bowel resection [6]. The delayed diagnosis and surgical treatment of appendicitis increases the risk of appendiceal perforation, which is associated with several challenges and complications and can lead to shock or death [4,7]. Perforated appendicitis has been reported to have a mortality rate of 0.2–0.8% [8]. Several diagnostic modalities—such as laboratory inflammatory markers, various scoring systems, abdominal computed tomography (CT), and abdominal ultrasonography (US)—have been used for the diagnosis of appendicitis. The current gold-standard diagnostic tool is abdominal CT [9,10].

Acute appendicitis is the most common cause of non-obstetric surgical disease in pregnant women [11,12]. It accounts for 65.6% of non-traumatic surgical emergencies in pregnant women [11]. It has a reported frequency of 1/700–1/4000 in pregnant women [13,14,15,16,17]. Acute appendicitis is most prevalent in the second trimester of pregnancy, and it causes the most complications during the second trimester [18].

There are several specific considerations regarding appendicitis in pregnant women. The symptoms of appendicitis in pregnant women can be similar to those in non-pregnant patients, but they may be masked by physiologic changes and obstetric problems during pregnancy. The diagnosis of appendicitis in pregnant women is made more challenging by the inability to use CT (because of the radiation hazard to the fetus), which is the most useful modality for diagnosing appendicitis [19,20]. The delayed diagnosis and appropriate treatment of appendicitis in pregnant women is associated with higher rates—and higher associated mortality rates (relative to the general population)—of complications such as amniotic fluid infection, pneumonia, and sepsis. These problems can lead to increased morbidity and mortality for both the mother and fetus [21,22].

Nowadays, the marriage rate and birth rate in South Korea have decreased significantly, with a birth rate of 0.78 in 2022. The age of women at their first marriage increased to 31.1 in 2022, and average age at first birth for women also increased to 32.3 in 2022 [23]. The age at first childbirth for women in South Korea has continued to increase from 27.5 years in 1995, to 31.3 years in 2010, and to 33.4 years in 2021. The infant mortality rate was 931/326,822 (2.8%) in 2018, 822/302,676 (8.7%) in 2019, and 674/272337 (2.5%) in 2020 [24]. The maternal mortality ratio per 100,000 was 11.3 in 2018, 9.9 in 2019, and 11.8 in 2020. In mothers over the age of 40, the maternal mortality ratio increased steeply [25]. Older pregnant women have a higher risk of complications during childbirth. The appropriate diagnosis and treatment of various diseases during pregnancy have become more important.

This study analyzed patients who underwent surgery, over a 10-year period, for acute appendicitis at a single tertiary medical institution in South Korea. We investigated the characteristics, diagnosis, and treatment of appendicitis in pregnant women in comparison with non-pregnant women.

## 2. Materials and Methods

### 2.1. Data Collection and Patients’ Grouping

We reviewed electronic medical records for acute appendicitis patients who underwent surgery at Inha University Hospital from January 2013 through December 2022. A total of 3532 patients who eventually underwent surgery for acute appendicitis visited the emergency room during the study period. There were 828 patients who were reproductive-aged women (between the ages of 15 and 44 years), as defined by the CDC (Centers for Disease Control and Prevention) [26].

Sixty-nine pregnant women with acute appendicitis were classified according to gestational age: 23 patients in the first trimester, 36 patients in the second trimester, and 10 patients in the third trimester.

We collected clinical data, including patients’ general characteristics (age, sex, and body mass index (BMI, kg/m^2^)); operation-related variables (operation time, surgical extent, drain use, histopathologic results, and American Society of Anesthesiologists (ASA) physical status classification); laboratory values (white blood cell count (WBC), hemoglobin concentration (Hb), absolute neutrophil count (ANC), and platelet count); postoperative clinical course; and postoperative surgical/medical complications.

### 2.2. Definitions

The *severity of appendicitis* was classified based on the postoperative histopathologic results. We reviewed the histopathologic data of all patients. Normal to mild inflammation, as well as suppurative and gangrenous appendicitis, were classified as uncomplicated appendicitis. Perforated appendicitis was classified as complicated appendicitis.

The *surgical extent* was divided into two categories based on the extent of surgery, regardless of whether the patient had an open or a laparoscopic approach. The appendectomy group included patients who only underwent appendectomy, and the extended surgery group included partial cecectomy, ileocecectomy, or right hemicolectomy.

*Negative appendectomy* was defined according to the postoperative biopsy result (normal to mild inflammation). Postoperative complication was defined as the occurrence of a relevant event related to surgery within 30 postoperative days.

### 2.3. Surgical Method

The surgical approach was defined according to the method used to perform surgery—open or laparoscopic. The choice of surgical approach was decided by the individual operating surgeon. All the patients were operated on as emergencies.

Open appendectomy: A 5 to 7 cm incision was made at the McBurney point. After the abdominal wall was opened, the appendix was identified. The peri-appendiceal tissue was dissected, and the appendiceal vessels and mesoappendix were ligated with absorbable suture material. The appendix base was ligated using the double tie method, and appendectomy was performed. The exposed mucosa of the appendix base was cauterized using a Bovie device. The appendix base stump was inverted using a pulse-string suture, and the operation was terminated.

Laparoscopic appendectomy: A 12 mm trocar was initially inserted at the infra-umbilicus or umbilicus. CO_2_ was insufflated at a pressure of 12 mmHg through an initial trocar, and two additional ports were inserted (5 mm at two sites; or 5 mm and 12 mm, depending on the surgeon’s preference). The appendix was identified using a laparoscope. The mesoappendix and appendiceal vessels were dissected using an ultrasonic shear device (Harmonic, Ethicon Endo-Surgery, Cincinnati, OH, USA). The appendix base was ligated using Endoloops (Vycril Endoloop-0, Ethicon Endo-Surgery) or polymetric clips (Hem-o-lok, Teleflex, Morrisville, NC, USA). The appendix was resected, and removal was performed. The appendix base mucosa tip was cauterized using the Bovie device, and the surgery was terminated.

Single-port laparoscopic appendectomy: A single port (Gloveport, Nelis, Bucheon, Gyeonggi-do, Republic of Korea) was used for laparoscopy instead of the traditional three ports. A 2 to 3 cm transumbilical incision was performed, and a single port was inserted through the transumbilical incision. The overall surgical procedure was conventional laparoscopic appendectomy.

### 2.4. Statistical Analysis

Statistical analysis was performed using SPSS Statistics for Windows, version 28.0 IBM Corp., Armonk, NY, USA). The chi-square test was used for cross-table analysis according to the sample size. Unpaired *t*-tests were used to compare the means between the two clinical groups. Pregnant women were classified into subgroups according to gestational age, and ANOVA (analysis of variance) was used in the subgroup analyses. Statistical significance was defined by *p* values < 0.05.

### 2.5. Ethics

The study protocol was approved by the Institutional Review Board of Inha University Hospital (IRB number: INH 2023-03-022).

## 3. Results

The 828 patients were reproductive-aged women between the ages of 15 and 44 years. There were 759 non-pregnant women and 69 pregnant women. The clinical characteristics are described in Table 1. The mean age and BMI were not significantly different between the two groups. The mean ASA class value representing the preoperative condition of the patients was significantly higher in the pregnant group (1.93 ± 0.53 versus 2.47 ± 0.53; *p* < 0.001), and the proportion of ASA class III cases (patients with severe systemic disease) was significantly higher in the pregnant group. There was no significant difference in operation time between the two groups, but the mean hospital stay was significantly longer in the pregnant group (3.80 ± 2.94 versus 6.36 ± 4.75; *p* < 0.001).

Table 2 shows the histopathologic and surgical characteristics of the non-pregnant group and the pregnant group. There was no difference between the two groups in the proportion of complicated appendicitis (59/759 (7.8%) versus 5/69 (7.2%); *p* = 0.875). There was no significant intergroup difference in the detailed histopathologic results. The negative appendectomy rate was higher in the pregnant group, but there was no significant difference in this regard (12/759 (1.6%) versus 3/69 (4.3%); *p* = 0.099). There was no significant difference in the proportions of extended surgery between the two groups (5/714 (0.7%) versus 1/69 (1.4%); *p* = 0.496). There were also no intergroup differences in intraoperative drain placement rates and the readmission rates for problems related to acute appendicitis within 30 days after surgery.

Table 3 shows the laboratory results before surgery and 2 days after surgery. In both groups, inflammatory markers, such as WBC and ANC, were increased. The mean WBC count of the pregnant group was significantly higher than that of the non-pregnant group (12,610 ± 4062/µL versus 14,160 ± 3851/µL; *p* = 0.003), and the mean ANC was also significantly higher in the pregnant group (10,281 ± 4010/µL versus 11,892 ± 4003/µL; *p* = 0.002). Laboratory tests were performed 2 days after surgery. In both groups, the WBC and ANC values decreased within the normal range. Even though inflammatory markers improved within the normal range, the mean WBC and ANC values of the pregnant group were significantly higher than those of the non-pregnant group. The preoperative and postoperative Hb levels were significantly lower in the pregnant group.

A total of 69 pregnant women were classified as follows: 23 patients in the first trimester, 36 patients in the second trimester, and 10 patients in the third trimester. Table 4 describes their clinical characteristics according to this subgroup classification. Women in the first-trimester subgroup were significantly younger than those in the other groups (28.22 ± 6.31 versus 32.61 ± 4.77 versus 31.60 ± 4.99; *p* = 0.011). There were no significant differences among the subgroups in terms of ASA classifications. Hospital stays were longer in the second-trimester subgroup relative to the other subgroups, but there was no significant difference in this regard. The mean operation time was statistically significantly longest in the third-trimester subgroup (62.22 ± 23.95 min versus 69.31 ± 38.12 min versus 104.40 ± 62.22 min, respectively; *p* = 0.024).

The histopathologic and surgical results of the three subgroups were reviewed (Table 5). There were no significant differences among the subgroups in rates of complicated appendicitis, negative appendectomy, or extended surgery. There were also no differences among the subgroups in terms of intraoperative drain placement rates or readmission rates within 30 days after surgery.

Table 6 shows the laboratory test results of the three subgroups before and after surgery. The preoperative WBC and ANC values were highest in the second-trimester subgroup, but there was no statistically significant difference in this regard. In the laboratory tests at 2 postoperative days, the WBC and ANC values were highest in the second-trimester subgroup, but this difference was not statistically significant. WBC and ANC recovered to within normal ranges in all three subgroups after surgery.

## 4. Discussion

It has been reported that acute appendicitis occurs in more than 1/20 of women of reproductive age [27]. In this study, acute appendicitis was diagnosed in 69 (8.8%) of 783 reproductive-aged women and 69 (4.3%) of all 1618 women. These results were higher than previous reports because patients with the most severe clinical features and diagnoses (such as acute appendicitis) are routinely referred to our hospital. In this study, pregnant women with appendicitis were most commonly in the second trimester, as in previous reports [18,28]. In our study, there were two cases of preterm labor and stillbirth due to appendicitis. Both of these cases occurred in pregnant women in their second trimesters, and the histopathology results revealed the presence of complicated appendicitis with perforation. The rate of complicated appendicitis was higher in this study than in other previously reported studies [29,30]. There was no significant difference between pregnant and non-pregnant women in this regard.

Because of the increase in subcutaneous fat and intra-abdominal fat, there are many limitations in obese acute appendicitis patients compared to non-obese acute appendicitis patients in the diagnosis using US [31,32]. Sauvain et al. reported that the usefulness of US is questionable in the diagnosis of appendicitis in overweight patients with a BMI > 25 kg/m^2^ [33]. It is known that the subcutaneous fat of women with normal weight before pregnancy is thinner than that of obese women before pregnancy. The prepregnancy overweight ratio of Korean women was 14.5–15.2% in 2017 [34]. The prepregnancy overweight ratio of American women was known to be 25.6% in 2014 [35]. Although the gestational age of Korean women is getting older compared to Western countries, the diagnosis of appendicitis in pregnant women using US can be performed more easily, quickly, and accurately in South Korea. It was thought that there was no significant difference in the rate of complicated appendicitis, extended surgery, or complications compared to non-pregnant appendicitis patients in this study.

Several studies have reported favorable outcomes associated with the non-operative treatment of uncomplicated acute appendicitis in the general population [3,4,5]. Matthew et al. performed a meta-analysis on the non-operative treatment of complicated appendicitis in pregnant women [30], and they found that the non-operative treatment of complicated appendicitis had a failure rate of 2646/3600 (73.5%). It has been reported that the failure of non-operative management increases maternal and fetal morbidity. Appendicitis is a risk factor for amniotic fluid infection, pneumonia, and sepsis in pregnant women [36,37]. The failure of non-operative treatment is associated with fetal morbidity and mortality, including in the contexts of preterm delivery, miscarriages, and stillbirths. Preterm labor in pregnancy-associated appendicitis has been reported to arise in 5–11% of cases. Perinatal mortality has been reported at 1.5% for uncomplicated appendicitis and 37% for perforated appendicitis [36,38]. In our study, preterm labor occurred in 2 (2.9%) of 69 patients with appendicitis and 2 (40.0%) of 5 patients with complicated appendicitis. All fetuses born after preterm labor died. If appendicitis is diagnosed in pregnant women, surgery should be strongly considered. The national guideline published by the Society of American Gastrointestinal and Endoscopic Surgeons also strongly recommends appendectomy for acute uncomplicated appendicitis in pregnant women [39].

In several previous studies, inflammatory markers, such as WBC and C-reactive protein levels, were found to be increased in healthy pregnant women [40,41,42]. Bazdar et al. reported appendicitis in pregnancy to be associated with WBC values over 18,000/µL (as a diagnostic parameter) [43]. Quinn et al. reported that a maternal relative neutrophil count <70% had a 100% negative predictive value for appendicitis [44]. In this study, pregnant women with appendicitis had significantly higher WBC and ANC values than non-pregnant women with appendicitis. However, WBC values did not reach 18,000/µL. The pregnant group had a mean relative neutrophil count of 83.9%, which was higher than the 81.5% determined for the non-pregnant group. WBC and ANC values normalized on the second postoperative day, but the mean WBC and ANC values of the pregnant group were significantly higher than those of the non-pregnant group. One study found that neutrocyte-to-lymphocyte ratios were higher in the second and third trimesters than in the first trimester [45]. In this study, there was no variation in the mean WBC or ANC according to gestational age. The WBC and ANC values were higher in the second trimester, but this was a non-significant trend.

The accurate diagnosis of appendicitis in pregnant women is very important. Accurate diagnosis can reduce complications and negative appendectomy. Additionally, delayed diagnosis can increase maternal and fetal mortality and morbidity [21,45]. Abdominal US is performed as the first option for pregnant women with appendicitis. The sensitivity of abdominal US has been reported to be 50–70%, with the specificity reported to be 83–100% [46,47]. In US, the diagnosis of appendicitis is based on the following criteria: the diameter of the appendix is >7 mm, and there is probe tenderness. Additionally, the echo signal is increased by inflammation in the surrounding tissue. Effusions or fluid collections are also commonly observed in the right fossa or Douglas fossa [12]. As the size of the uterus increases in the second and third trimesters, it is difficult to find the appendix as it is displaced by the uterus (highly situated and more lateral). Low-dose CT can be used, but the radiation hazard to the fetus should be considered. If the mother and fetus are stable, abdominal magnetic resonance imaging (MRI) can be a helpful diagnostic method. The diagnostic accuracy of MRI for acute appendicitis has been reported to be 90.4% (positive predictive value) and 99.5% (negative predictive value) [40,47]. In this study, three patients were diagnosed with unidentified appendicitis with a clinical suspicion of appendicitis in US, and they were accurately diagnosed with acute appendicitis using abdominal MRI. Several studies have reported negative appendectomy rates of 13–38% [41,48,49]. Abdominal MRI can be used to reduce the rate of negative appendectomy for pregnant women. Additionally, low-dose abdominal CT and abdominal MRI can be helpful to patients who are not diagnosed via US but highly suspicious of appendicitis.

There are several concerns about laparoscopic appendectomy in pregnant women. The appendix is displaced from its normal position from the first to the third trimester. The operative field and adequate space cannot be properly secured due to the enlarged uterus. There is a high possibility of uterine and intra-abdominal organ injury during trocar insertion. Moreover, the effect of a pneumatic pressure of 12 mmHg is still unclear. Despite these concerns, many studies have reported that laparoscopic results are better than those associated with open appendectomy [28,36,39,50,51]. Laparoscopy has the advantage of the easier exploration of areas that cannot be directly visualized through a camera and has the advantage of exploring various variant locations [37]. Surgery remains the mainstay of treatment for appendicitis in pregnant women, and laparoscopic surgery is preferred over open surgery due to its lower risk of complications. In this study, appendectomy was associated with similar outcomes in the pregnant and non-pregnant groups. Laparoscopic appendectomy was performed at a similar rate to open appendectomy in all pregnant women. There was no intergroup difference in postoperative results. However, the operative time of the third-trimester subgroup was significantly longer than that of the first- and second-trimester subgroups.

Previous research found that abortion and threatened preterm labor could be prevented when a tocolytic agent was administered systemically for uterine contraction prevention [52]. In our study, tocolytics were used in 6 out of 69 patients, and preterm labor occurred in 2 patients. Considering that appendicitis in pregnancy is most common in the second trimester, that the fetus is still immature in the second trimester, and that the probabilities of stillbirth and fetal sequelae are high, it is necessary to actively manage tocolytics in acute appendicitis in pregnancy. The management of appendicitis in pregnancy involves a multidisciplinary approach, with close collaboration between obstetricians, general surgeons, and anesthesiologists.

### Limitations

This was a retrospective study, and we cannot rule out selection bias. Although 10 years of medical records were reviewed, the number of patients in the pregnant group was insufficient compared to the meta-analysis. However, the number of pregnant patients in this study was not small compared to other single-center analyses. We evaluated the clinical characteristics of pregnant women with appendicitis compared to non-pregnant women of childbearing age. In our hospital, we performed an appendectomy according to duty. Non-specialized surgeons, including breast or thyroid surgeons, performed appendectomy without discrimination. Our results do not apply to specialized gastrointestinal surgeons but to generalized surgeons regarding appendectomy for pregnant women. Since this is a single-center study, although a comparison can be made with other single-center studies, the results may not be generalizable.

## 5. Conclusions

Surgery is the gold-standard treatment for pregnant women with appendicitis. Given that morbidity and mortality are high in pregnant women, immediate surgical treatment should be strongly considered. Pregnant women with appendicitis have several particular features that differ from those of the general population, and special attention should be paid to obstetric complications. To prevent undiagnosed appendicitis or negative appendectomy, more accurate diagnosis using abdominal MRI should be considered.

## Figures and Tables

**Table 1:** Clinical characteristics of the reproductive-aged women with appendicitis.

Variables	All (*n* = 828)	Non-Pregnant Appendicitis (*n* = 759)	Pregnant Appendicitis (*n* = 69)	*p*-Value
**Age (years, mean ± sd)**	29.99 ± 8.27	29.90 ± 8.56	31.00 ± 5.65	0.288
**Gender**				
Female	828 (100.0%)	759 (100.0%)	69 (100.0%)	
**ASA class**	1.97 ± 0.55	1.93 ± 0.53	2.47 ± 0.53	<0.001
ASA I	130 (15.7%)	130 (17.1%)	0 (0.0%)	<0.001
ASA II	554 (66.9%)	516 (68.0%)	38 (55.0%)	
ASA III	107 (12.9%)	77 (10.3%)	30 (43.5%)	
ASA IV	1 (0.1%)	0 (0.0%)	1 (1.5%)	
Value not being recorded	36 (4.4%)	36 (4.7%)	0 (0.0%)	
**BMI (kg/m^2^)**	23.03 ± 14.39	23.01 ± 4.97	23.23 ± 3.66	0.907
**Operation time (minutes, mean ± sd)**	72.48 ± 92.55	72.43 ± 95.91	73.03 ± 40.23	0.959
**Hospital stay (days, mean ± sd)**	4.02 ± 3.20	3.80 ± 2.94	6.36 ± 4.75	<0.001

ASA: American Society of Anesthesiologists. ASA I: a normal healthy patient. ASA II: a patient with mild systemic disease. ASA III: a patient with severe systemic disease. ASA IV: a patient with an incapacitating systemic disease that is a constant threat to life. Value not being recorded: no ASA score in medical records. BMI: body mass index, kg/m^2^. NA: not applicable.

**Table 2:** Histopathologic and surgical characteristics of the reproductive-aged women with appendicitis.

Pathologic Result	All (*n* = 828)	Non-Pregnant Appendicitis (*n* = 759)	Pregnant Appendicitis (*n* = 69)	*p*-Value
**Uncomplicated appendicitis**	764 (92.3%)	700 (92.2%)	64 (92.8%)	0.875
**Complicated appendicitis (perforated)**	64 (7.7%)	59 (7.8%)	5 (7.2%)	
**Biopsy result details**				
Normal to mild inflammation	15 (1.8%)	12 (1.6%)	3 (4.3%)	0.322
Suppurative	685 (82.7%)	632 (83.3%)	53 (76.8%)	
Gangrenous	63 (7.6%)	55 (7.2%)	8 (11.6%)	
Perforated	64 (7.7%)	59 (7.8%)	5 (7.2%)	
Tumor	1 (0.1%)	1 (0.1%)	0 (0.0%)	
**Negative appendectomy**	15/828 (1.8%)	12/759 (1.6%)	3/69 (4.3%)	0.099
**Surgical extent**				
Appendectomy	822 (99.3%)	754 (99.3%)	68 (98.6%)	0.459
Extended surgery	6 (0.7%)	5 (0.7%)	1 (1.4%)	
**Surgical method**				NA
Open appendectomy	165 (19.9%)	147 (19.4%)	18 (26.1%)	
Laparoscopic appendectomy	465 (79.9%)	607 (80.0%)	50 (72.5%)	
Cecum wedge resection (partial cecectomy)	3 (0.4%)	2 (0.3%)	1 (1.4%)	
Others	3 (0.3%)	3 (0.4%)	0 (0.0%)	
**Intraoperative drain placement**				
No	597 (72.1%)	547 (72.1%)	50 (72.5%)	0.944
Yes	231 (27.9%)	212 (27.9%)	19 (27.5%)	
**Readmission in 30 days**				
No	809 (98.9%)	742 (99.1%)	67 (97.1%)	0.135
Yes	9 (1.1%)	7 (0.9%)	2 (2.9%)	
**Readmission cause**				
Post OP ileus	4	3	1	
Post OP pain	0	0	0	
Post OP fluid collection	4	4	0	
Wound problem	1	0	1	

NA: not applicable. OP: operation.

**Table 3:** Laboratory test results of the reproductive-aged women with appendicitis.

Laboratory Test	All (*n* = 828)	Non-Pregnant Appendicitis (*n* = 759)	Pregnant Appendicitis (*n* = 69)	*p*-Value
**Preoperative period**				
WBC	12,870 ± 4088	12,760 ± 4091	14,160 ± 3851	0.008
Hb	12.95 ± 1.22	13.03 ± 1.18	11.99 ± 1.25	<0.001
ANC	10,532 ± 4062	10,411 ± 4048	11,892 ± 4003	0.005
PLT	255,350 ± 59,938	255,710 ± 60,338	251,340 ± 55,523	0.574
**Postoperative (2 days)**				
WBC	6870 ± 2337	6670 ± 2194	9330 ± 2577	<0.001
Hb	10.79 ± 1.16	10.83 ± 1.14	10.32 ± 1.32	0.001
ANC	4754 ± 2298	4533 ± 2127	7441 ± 2601	<0.001
PLT	212,820 ± 53,598	211,640 ± 53,559	226,860 ± 52,493	0.036

WBC: white blood cell count (/µL). Hb: hemoglobin (g/dL). ANC: absolute neutrophil count (/µL). PLT: platelet count (/µL).

**Table 4:** Clinical characteristics of the pregnant women with appendicitis.

Variables	1st Trimester (*n* = 23)	2nd Trimetster (*n* = 36)	3rd Trimetster (*n* = 10)	*p*-Value
**Age (years, mean ± sd)**	28.22 ± 6.31	32.61 ± 4.77	31.60 ± 4.99	0.011
**ASA class**	2.43 ± 0.51	2.51 ± 0.56	2.40 ± 0.52	0.777
ASA I	0 (0.0%)	0 (0.0%)	0 (0.0%)	0.639
ASA II	13 (56.5%)	19 (51.4%)	6 (60.0%)	
ASA III	10 (43.5%)	16 (45.7%)	4 (40.0%)	
ASA IV	0 (0.0%)	1 (2.9%)	0 (0.0%)	
**Pregnancy weeks**	8.74 ± 3.80	20.25 ± 3.48	33.20 ± 2.25	<0.001
**BMI (kg/m^2^)**	23.35 ± 4.08	22.33 ± 3.04	26.01 ± 3.49	0.017
**Operation time (minutes, mean ± sd)**	65.22 ± 23.95	69.31 ± 38.12	104.40 ± 62.23	0.024
**Hospital stay (days, mean ± sd)**	5.70 ± 3.09	6.94 ± 5.79	5.80 ± 3.74	0.574
**Abdomen US in our hospital**	13/23 (43.5%)	18/36 (50.0%)	6/10 (60.0%)	0.678
**Abdomen MR in our hospital**	0/23 (0.0%)	2/36 (5.6%)	1/10 (6.5%)	0.210

ASA: American Society of Anesthesiologists. ASA I: a normal healthy patient. ASA II: a patient with mild systemic disease. ASA III: a patient with severe systemic disease. ASA IV: a patient with an incapacitating systemic disease that is a constant threat to life. BMI: body mass index, kg/m^2^. NA: not applicable.

**Table 5:** Histopathologic and surgical characteristics of the pregnant women with appendicitis.

Pathologic Result	1st Trimester (*n* = 23)	2nd Trimetster (*n* = 36)	3rd Trimetster (*n* = 10)	*p*-Value
**Uncomplicated appendicitis**	22 (95.7%)	34 (94.4%)	8 (80.0%)	0.884
**Complicated appendicitis (perforated)**	1 (4.3%)	2 (5.6%)	2 (20.0%)	
**Biopsy result details**				
Normal to mild inflammation	2 (8.7%)	1 (2.8%)	0 (0.0%)	0.384
Suppurative	16 (69.6%)	29 (80.6%)	8 (80.0%)	
Gangrenous	4 (17.4%)	4 (11.1%)	0 (0.0%)	
Perforated	1 (4.3%)	2 (5.6%)	2 (20.0%)	
**Negative appendectomy**	2/23 (8.7%)	1/35 (2.8%)	0/10 (0%)	0.425
**Surgical extent**				
Appendectomy	23 (100.0%)	35 (97.2%)	10 (100.0%)	0.628
Extended surgery	0 (0.0%)	1 (2.8%)	0 (0.0%)	
**Surgical method**				0.736
Open appendectomy	5 (21.7%)	9 (25.0%)	4 (40.0%)	
Laparoscopic appendectomy	18 (78.3%)	26 (72.2%)	6 (60.0%)	
Cecum wedge resection (partial cecectomy)	0 (0.0%)	1 (2.8%)	0 (0.0%)	
**Intraoperative drain placement**				
No	17 (73.9%)	28 (77.8%)	5 (50.0%)	0.216
Yes	6 (26.1%)	8 (22.2%)	5 (50.0%)	
**Readmission in 30 days**				
No	23 (100.0%)	34 (94.4%)	10 (100.0%)	0.389
Yes	0 (0.0%)	2 (5.6%)	0 (0.0%)	
**Readmission cause**				
Post OP ileus	0	1	0	
Post OP pain	0	0	0	
Post OP fluid collection	0	0	0	
Wound problem	0	1	0	

NA: not applicable. OP: operation.

**Table 6:** Laboratory test results of the appendicitis pregnant women.

Laboratory Test	1st Trimester (*n* = 23)	2nd Trimetster (*n* = 36)	3rd Trimetster (*n* = 10)	*p*-Value
**Preoperative period**				
WBC	13,460 ± 3962	14,690 ± 3903	13,750 ± 3366	0.483
Hb	12.44 ± 1.29	11.82 ± 0.92	11.48 ± 1.99	0.085
ANC	11,066 ± 4195	12,535 ± 4078	11,352 ± 2889	0.376
PLT	248,550 ± 63,275	257,940 ± 52,817	230,120 ± 43,222	0.430
**Postoperative (2 days)**				
WBC	8660 ± 2851	9880 ± 2141	8860 ± 3236	0.226
Hb	10.86 ± 1.59	10.01 ± 0.91	10.16 ± 1.65	0.074
ANC	6437 ± 2636	8198 ± 2237	6770 ± 3190	0.051
PLT	234,000 ± 63,754	228,840 ± 43,103	201,380 ± 54,137	0.322

WBC: white blood cell count (/µL). Hb: hemoglobin (g/dL). ANC: absolute neutrophil count (/µL). PLT: platelet count (/µL).

## Data Availability

No new data were created or analyzed in this study. Data sharing is not applicable to this article.
